# The Intricate Role of Non-Coding RNAs in Sepsis-Associated Disseminated Intravascular Coagulation

**DOI:** 10.3390/ijms24032582

**Published:** 2023-01-30

**Authors:** Irene Cánovas-Cervera, Elena Nacher-Sendra, Rebeca Osca-Verdegal, Enric Dolz-Andrés, Jesús Beltrán-García, María Rodríguez-Gimillo, Carolina Ferrando-Sánchez, Nieves Carbonell, José Luis García-Giménez

**Affiliations:** 1 Department of Physiology, Faculty of Medicine and Dentistry, University of Valencia, 46010 Valencia, Spain; 2 Health Research Institute INCLIVA, 46010 Valencia, Spain; 3 Center for Biomedical Research Network on Rare Diseases (CIBERER), Carlos III Health Institute, 46010 Valencia, Spain; 4 Department of Medicine, Division of Regenerative Medicine, University of California, San Diego, CA 92093, USA; 5 Intensive Care Unit, Clinical University Hospital of Valencia, 46010 Valencia, Spain

**Keywords:** non-coding RNAs, miRNAs, lncRNAs, biomarkers, sepsis, Disseminated Intravascular Coagulation

## Abstract

Disseminated Intravascular Coagulation (DIC) is a type of tissue and organ dysregulation in sepsis, due mainly to the effect of the inflammation on the coagulation system. Unfortunately, the underlying molecular mechanisms that lead to this disorder are not fully understood. Moreover, current biomarkers for DIC, including biological and clinical parameters, generally provide a poor diagnosis and prognosis. In recent years, non-coding RNAs have been studied as promising and robust biomarkers for a variety of diseases. Thus, their potential in the diagnosis and prognosis of DIC should be further studied. Specifically, the relationship between the coagulation cascade and non-coding RNAs should be established. In this review, microRNAs, long non-coding RNAs, and circular RNAs are studied in relation to DIC. Specifically, the axis between these non-coding RNAs and the corresponding affected pathway has been identified, including inflammation, alteration of the coagulation cascade, and endothelial damage. The main affected pathway identified is PI3K/AKT/mTOR axis, where several ncRNAs participate in its regulation, including miR-122-5p which is sponged by circ_0005963, ciRS-122, and circPTN, and miR-19a-3p which is modulated by circ_0000096 and circ_0063425. Additionally, both miR-223 and miR-24 were found to affect the PI3K/AKT pathway and were regulated by lncGAS5 and lncKCNQ1OT1, respectively. Thus, this work provides a useful pipeline of inter-connected ncRNAs that future research on their impact on DIC can further explore.

## 1. Introduction

Sepsis is a life-threatening condition that arises when the host’s immune response to infection damages its own tissues and leads to organ failure [1]. Despite advances in therapeutic approaches and treatments applied in Intensive Care Units (ICU) worldwide, sepsis remains a serious concern in all healthcare systems affecting more than 48 million people every year. It continues to be the leading cause of death from infection, accounting for more than 11 million deaths annually [2], which is expected to increase in the coming years due to the generation of antibiotic resistance and the aging of the population, among other factors.

The characteristic immune dysregulation of sepsis promotes severe endothelial cell dysfunction, which leads to a perturbation of endothelial hemostasis and produces vascular reactivity [3]. Additionally, it promotes activation of the coagulation cascade, causes tissue edema, and compromises the perfusion of vital organs [4]. This critical feature of sepsis is considered to play a major role in the pathophysiology of systemic inflammation, contributing to the advancement of organ failure during sepsis [5]. This is of special relevance since severe endothelial injury can expose the vascular basement membrane and promote the expression of procoagulant and fibrinolytic molecules in endothelial cells [6]. Importantly, critical hemostatic changes related to blood coagulation occur during sepsis, which contribute to microvascular thrombosis and acute disseminated intravascular coagulation (DIC). 

DIC is characterized by a strong, generalized, microvascular thromboembolism and the perturbation of the endothelium. Consequently, endothelial cells produce a repertoire of procoagulant and fibrinolytic molecules, consume platelets, and active the coagulation cascade, resulting in clotting activation and fibrin deposition at the site of vascular damage [6], ultimately causing hemorrhagic manifestations [7]. Critically, sepsis-associated DIC leads to organ dysfunction [7] and remains a serious complication of sepsis that increases the risk of death in septic patients.

### 1.1. Key Mediators and Factors Regulating Coagulation

The early identification of DIC can help to anticipate the molecular events contributing to endothelial dysfunction and thrombosis. This prediction helps to decide the appropriate treatments. Currently, the early diagnosis of DIC is currently a challenge because it is not easy and usually requires the determination of some combination of basic coagulation markers and the experience of the clinician [8,9]. The main clinical feature linked to DIC is thrombocytopenia and some laboratory markers associated with DIC, mainly consisting of elevated levels of fibrin-related markers, blood fibrinogen concentration, elevated antithrombin activity, increased concentration of thrombin–antithrombin complex, the presence of prothrombin fragments, and elevated consumption of coagulation factors. In fact, a hallmark of DIC is the combination of platelet depletion, increased fibrinogen/fibrin degradation products such as D-Dimer, and a prolonged prothrombin time (PT) [8,9,10,11]. 

This approach does not provide an efficient tool for DIC identification, so the immediate consequence is the low specificity of such diagnoses. Thus, the ability to discriminate DIC from other thrombocytopenic diseases is essential in the appropriate management of septic patients [12]. Therefore, research has focused on the use of other biomarkers with the potential to identify those patients with a high risk of developing DIC or markers to assess DIC severity, such as protein C and, particularly, activated protein C (APC). APC performs an anticoagulation function by reducing thrombin formation and inactivating coagulation factors Va and VIIIa. APC not only exhibits anticoagulant properties but also has demonstrated anti-inflammatory, anti-apoptotic, and neuroprotective activities and protection of endothelial barrier function [13]. In this regard, low APC levels correlated with the severity of the disease in patients with sepsis and DIC: the lower the amount of APC, the greater the severity of the disease [10,14].

Vascular endothelium homeostasis has been identified as a key player in the fibrinolysis process since endothelial cells produce, regulate, and bind fibrinolytic proteins related to the coagulation process. Importantly, the vascular endothelial growth factor (VEGF) has been studied in endothelium homeostasis due to its role in the permeability of endothelial cells and its function in regulating cell proliferation and migration in endothelial cells, among others [15]. VEGF activates the expression of tissue plasminogen activator (tPA), which is produced in the endothelium, urokinase plasminogen activator, plasminogen activator inhibitor-1 (PAI-1), and the receptor for the urokinase plasminogen activator, which is a key process during the extrinsic and intrinsic coagulation pathway. In addition, the fibrinolysis process may be affected by VEGF, whose low expression reduces the expression of tPA and PAI-1 [16,17,18].

Several molecular pathways are altered due to DIC, and therefore could be used to both diagnose and monitor the progression of patients. Consequently, it is interesting to study these factors concerning other useful biomarkers such as non-coding RNAs.

### 1.2. Non-Coding RNAs Involved in Disseminated Intravascular Coagulation

Current knowledge indicates that the human genome consists of only 1.5% coding sequences while 98% is non-coding regions [19]. Non-coding RNAs (ncRNAs) are transcripts located in non-coding DNA regions [20]. They are characterized as having a high stability in different biospecimens, high specificity, and reproducibility when they are analyzed with a wide array of molecular tools and are therefore postulated as robust biomarkers in many pathologies [19].

In recent years, several subtypes of ncRNAs have emerged as regulators of gene and protein expression by influencing messenger RNA (mRNA) function, DNA methylation, or histone modification [19,20,21]. Depending on the length of the ncRNA after transcription, ncRNAs are classified as long ncRNAs (lncRNAs) with more than 200 base pairs, and small ncRNAs with fewer than 200 base pairs. Although some ncRNAs were identified in the pregenomic era, the interest in exploring the potential of these intriguing molecules did not start until the 2000s. 

MicroRNAs (miRNAs) are small single-stranded ncRNA molecules between 20 and 23 nucleotides in length and are generally found inside the cell and in body fluids as cell-free miRNAs, bound to proteins (e.g., coupled to Ago2 protein) or contained in exosomes [22,23]. They perform regulatory functions of gene expression modulating relevant physiological processes and play a key role in severe tissue injuries. In general, miRNAs target their complementary mRNAs through the 3′-untranslated regions (UTR) [24]. To a lesser extent, miRNAs have also been shown to perform an inhibitory function by binding to the coding region or the 5′-UTR of target mRNAs. In this way, they regulate protein translation by repressing translation through the sequestering or degradation of the target mRNA. However, one-third of human RNAs have been postulated to have one or more miRNA binding sites. Furthermore, one miRNA may bind to multiple targets. Therefore, miRNA regulation is a complex web that ensures the correct functioning of molecular processes [24].

Recently, it has been discovered that miRNAs are involved in the regulation of many complex molecular mechanisms, as well as influencing physiological and clinical conditions. As a result, they are used as clinical biomarkers, in both diagnosis and prognosis, having a direct implication in the pathways dysregulated due to a pathology [24]. In this review, we will focus on the hemostatic system, specifically on coagulation. Although little is known about the role of miRNA networks in vascular processes, such as hemostatic abnormalities and coagulopathy [25,26,27], it has been described that coagulation factors, such as fibrinogen [28] and tissue factor (TF) [27,29], interact with miRNAs, which may have an impact on hematological pathologies.

On the other hand, lncRNAs are similar of more than 200 base pairs and are similar in structure to mRNA [30]. They are found in the cell nucleus and cytoplasm and participate in DNA methylation and chromatin modification, among others. Today, almost 53,000 different lncRNAs have been described, although only 1000 of them can be justified by their functional importance [31].

A subtype of lncRNAs is circular RNAs (circRNAs) which are characterized by having a circular shape created from noncanonical splicing of mRNA. The 3′ and 5′ ends are interconnected to form a continuous covalently closed loop, which enables their circular structure [32]. This structure prevents their degradation, as RNA exonucleases cannot easily access this circular structure [31]. CircRNAs are generally found in the cell cytoplasm or stored in exosomes [33] and are mainly derived from protein-coding genes, but are not produced by the normal RNA splicing model.

CircRNAs are widely expressed in mammalian tissues and cells, with a high conservation rate between mammalian species [34]. Several studies have discussed about their high involvement and association in physiological and pathological processes [35,36,37], as well as their high stability and high abundance in body fluids. Thus, they have been proposed as potent biomarkers for a wide range of pathologies, including sepsis [38].

It should be noted that circRNAs have a dual role as therapeutic agents: through their ability to modulate the response of other regulatory RNAs, such as miRNAs, and by binding to receptors located in the cell membrane [38], which is of great importance for further research into new ways to improve the diagnosis and prognosis of pathologies. Recently, it has been observed that several circRNAs function as miRNA “sponges”, where they can sequester specific miRNAs, thus modulating gene expression and transcriptional regulation. In addition, circRNAs have also been shown to modulate gene expression by interacting with RNA-binding proteins (RBPs) and even regulating translation directly [39,40,41]. Likewise, circRNAs are believed to be involved in the regulation of the inflammatory response. Numerous studies have shown that some circRNAs are associated with inflammation in traumatic injury models [32].

Having established the impact that ncRNAs and their subtypes have on the regulation of coagulation and inflammatory processes, the purpose of this study is to provide a map of their interactions with DIC-related dysregulated pathways. We aim to establish a comprehensive review of current knowledge and provide a possible future research focus to further advance DIC biomarkers.

### 1.3. Key Components in the Coagulation Cascade

**Figure 1:**
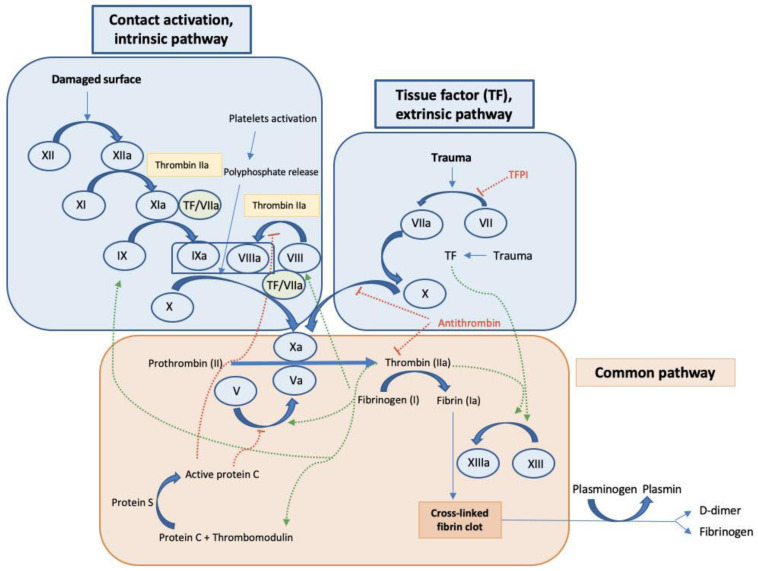
General coagulation cascade. Intrinsic pathway, extrinsic pathway, and common pathway. The cascade is initiated by surface contact or tissue damage leading to the activation of factor XII in the intrinsic pathway or factor VII in the extrinsic pathway, which both continues with the activation of the coagulation cascade leading to the activation of factor X and initiating the mechanisms included in the common pathway resulting in fibrin activation and finally clot formation. In addition, activating signals (green arrows), and inhibitory mechanisms (red dotted lines) are observed.

Here, we briefly describe the main factors participating in the intrinsic, extrinsic, and common pathways of the coagulation cascade (Figure 1), focusing on those factors that throughout this manuscript we describe can be regulated by some ncRNAs.

As is commonly known, factor XIII, or fibrin stabilizing factor in the common pathway, is activated by thrombin to generate factor XIIIa, which in turn participates in the crosslinking process of fibrin, therefore contributing to the stabilization of the blood clot [42]. Importantly, factor XIII levels in the plasma were significantly decreased in overt DIC compared to non-overt DIC patients and a significant inverse correlation was found between DIC scores and factor XIII activity. Moreover, factor XIII activity significantly correlated with other hemostatic markers including platelet count, PT, activated partial thromboplastin time (aPTT), fibrinogen, and D-Dimer [43]. Following the proteolytic cascade in the intrinsic pathway of coagulation, factor IX cleaves and activates factor X, which results in the conversion of prothrombin into thrombin, which is required to transform fibrinogen into fibrin in the final process of the common pathway. Factor X is also produced via the extrinsic pathway. Current studies consider TF, a transmembrane glycoprotein expressed in perivascular tissue, to be the main initiator of the extrinsic pathway, and thus, it is the initiator of the formation of blood clotting [44]. In the extrinsic pathway the tissue factor pathway inhibitor (TFPI) also participates as an anticoagulant protein that blocks the initiation of blood coagulation by inhibiting TF–Factor VIIa interaction. In these mechanisms, TF forms a complex with factor VIIa to initiate clotting through the activation of factor IX in the intrinsic pathway, which finally converges in the common pathway.

All these proteolytic cascades can act together to further facilitate the formation of the fibrin clot, thus promoting coagulation (Figure 1) [45].

The coagulation process is also affected by other proteins produced by platelets and endothelial cells with relevant roles in hemostasis. 

As described in the previous section, DIC is promoted by platelet consumption and microvascular thrombosis. This is facilitated by the release by endothelial cells of ultra-large von Willebrand factor (vWF) by endothelial cells, which in turn participate in platelet adhesion and consumption [46]. In addition, vWF and factor VIII can form a complex. vWF and factor VIII are two distinct, but related glycoproteins directly involved in the coagulation process. The vWF captures platelets thereby promoting platelet adhesion to thrombogenic surfaces as well as platelet-to-platelet cohesion during thrombus formation in the sites of vascular damage; also, vWF is the carrier for factor VIII in plasma contributing to reducing its degradation and clearance [47]. Factor VIII also accelerates the activation of factor X by activated factor IX in the common pathway of the coagulation cascade [48].

**Figure 2:**
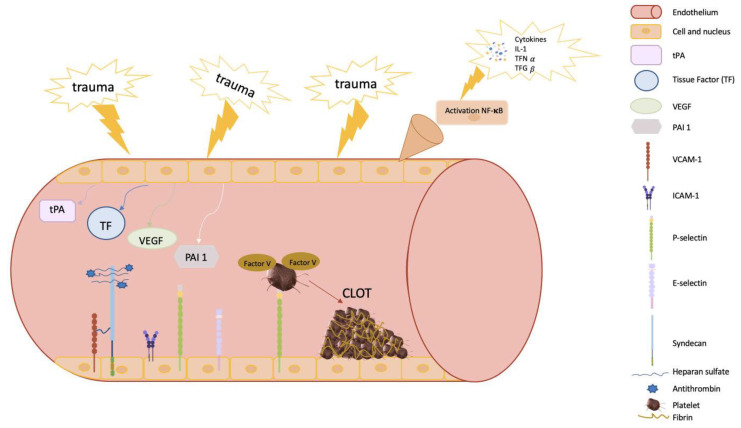
Diagram of the main components of endothelial vessels involved in clot formation. The endothelium plays a key role in coagulation. First, following trauma and injury to the endothelium, it contributes to platelet aggregation and recruitment of cells that participate in the conversion of fibrinogen to fibrin, forming a network and ultimately creating the clot. In addition, the endothelium is closely related to the PI3K/AKT pathway and endothelial factors (VCAM and ICAM). VCAM-1 contributes to the activation of PI3K. Moreover, the endothelium can activate antithrombin III, which is bound to syndecan by binding antithrombin to the endothelial surface and increasing the inhibitory action on the coagulation cascade. Inflammation is initiated by the release of TFN-α, triggering a series of molecules—selectins, VCAM, and ICAM—to recruit lymphocytes. VEGF can increase VCAM expression in endothelial cells. Vascular endothelial homeostasis is an important contributor to the process of fibrinolysis. VEGF, vascular endothelial growth factor, activates the expression of tissue plasminogen activator (tPA) and plasminogen activator inhibitor-1 (PAI-1). In addition, adhesion molecules (VCAM-1 and ICAM-1) expressed by the endothelial cells and released into the blood contribute to platelet adhesion, activation, and aggregation, and may activate procoagulant factors which, in turn, also contribute to platelet adhesion and aggregation.

Importantly, during sepsis, platelets can increase P-selectin expression, and expose on their surface phosphatidylserine, factor V, and its activated form on their surface, which are key mediators of the extrinsic pathway of coagulation. P-selectin further helps to adhere platelets to the endothelium and leukocytes, contributing to the increase in TF expression which altogether enhances thrombin generation [9,49] (Figure 2).

Therefore, the endothelium is also a key element participating in the coagulation process. In this regard, it is widely known that the inflammatory response during sepsis can initiate amplification of tissue damage affecting endothelial cells, contributing to local vasoconstriction at the endothelial level and limiting blood flow in the injured area [3,50,51]. Moreover, cell adhesion molecules (CAMs), such as vascular cell adhesion protein 1 (VCAM-1) and intercellular adhesion molecule 1 (ICAM-1), expressed by endothelial cells and released into the blood, contribute to platelet adhesion, activation, and aggregation, and can activate procoagulant factors which in turn also contribute to platelet adhesion and aggregation (Figure 2) [52].

In addition, endothelial injury causes exposure of the TF present in the fibroblasts of the endothelium, initiating the coagulation process, as described above [3,50,51], to finally convert fibrinogen into fibrin fibers and create a mesh around the platelet plug trapping platelets, blood cells, and plasma to carry out the formation of the fibrin clot [3,50,51,53,54].

Another interesting function of the endothelium is its ability to activate antithrombin III through the exposure of a glycosaminoglycan heparan sulfate (which simulates heparin). This molecule binds antithrombin to the endothelial surface and increases the inhibitory action on the coagulation cascade (Figure 2) [55].

Molecularly, the phosphatidylinositol 3-kinase (PI3K) and protein kinase B (AKT) pathway are involved in endothelial functions such as the regulation of vascular tone, angiogenesis, control of adhesion, and recruitment of leucocytes on the surface of the endothelial cell [56]. Here, it is important to stress that in endothelial cells, the PI3K/AKT pathway is the downstream activation of several receptors, including G-protein-coupled receptors (e.g., chemokine receptors), cytokine receptors (e.g., receptor for interleukin (IL)-8), tyrosine kinases (e.g., receptors for VEGF), integrins, and cell death receptors (e.g., tumor necrosis factor (TNF)-α receptor). Interestingly, PI3K appears to affect the function and the spatial distribution of E-selectin [57], therefore contributing to E-selectin-dependent neutrophil-endothelial interaction and neutrophil rolling and migration on endothelial cells in inflamed tissue [58]. In addition, the intriguing role of the endothelium in coagulation goes beyond its role in contributing to platelet aggregation and recruitment of cells that facilitate the formation of fibrin fibers and finally the clot. The PI3K/AKAT pathway and endothelial adhesion factors, such as VCAM, are inter-connected. Furthermore, it has been described how VCAM-1 contributes to the activation of PI3K [59,60].

## 2. The Intricate Pathways Modulated by Non-Coding RNAs in DIC

### 2.1. The Role of microRNAs in DIC

MiRNAs have been proposed as potential biomarkers for several pathologies since they play a key role in different molecular processes and thus can potentially determine the affected pathways due to diseases [23]. Moreover, miRNAs are stable in the cell and in exosomes, and thus are found in several body fluids which makes them easily accessible for measuring them [61]. Consequently, on-going research has studied the potential of miRNAs to improve both diagnosis and prognosis in several diseases, including trauma-induced coagulopathy and sepsis.

The inflamma-miR miR-155 was found to be upregulated the cardiac tissue of mice treated with lipopolysaccharides (LPS) [62] and in plasma samples from septic patients. These authors showed the potential use of circulating miR-155 to identify septic patients with cardiac injury from septic patients without cardiac injury, obtaining an AUC (area under the curve) of 0.863 in the receiver operating curve (ROC) [62]. Importantly, overexpression of the inflamma-miR miR-155 has proven to induce spontaneous endothelial damage and alteration of the endothelial barrier function, thus leading to vascular leakage in a claudin-1-dependent manner in several models of endotoxemia [63]. In addition, recent research has proposed miR-155 as a master regulator of the coagulation factor XIII in several diseases [64]. As described in the previous section, factor XIII participates in the crosslinking process of fibrin, stabilizing the blood clot. Therefore, these interesting findings provide an intriguing question about the possibility of modulating the expression of miR-155 to control the expression of factor XIII in patients who are suspected to have DIC.

One of the most-studied miRNAs in sepsis is miR-122, identified as a diagnostic and prognostic biomarker in the serum of patients with sepsis [65,66]. Specifically, miR-122-5p has been associated with coagulation abnormalities related to sepsis [67]. Wang et al. described an overexpression of miR-122-5p in abnormal coagulation patients. Additionally, coagulation-related parameters such as aPTT, fibrinogen, and antithrombin III were correlated with the level of miR-122 [67], so it can be stated that miR-122 levels correlate with non-normal and worrisome coagulation during sepsis [67]. Particularly, results suggest that miR-122 may regulate antithrombin III expression [67], thus affecting the anticoagulant and anti-inflammatory properties of antithrombin III. In other studies, miR-122 levels in septic patients were analyzed in both surviving and deceased patients and it was observed that miR-122 was overexpressed in non-surviving septic patients [65]. Wang et al. performed a similar study where they also differentiated between patients with normal and non-normal coagulopathy [67]. It was shown that miR-122 levels were significantly higher in the group of patients with non-normal coagulopathy. Moreover, Wang et al. demonstrated that miR-122 levels in patients with sepsis and non-normal coagulation had elevated miRNAs up to 13 days after admission, so miR-122 could be used as a coagulation index for the detection of coagulation disorders in patients with sepsis [67].

Ma et al. have shown the role of miR-125b in sepsis-induced cardiomyopathy [68] and how its overexpression in mouse hearts improved survival by improving heart function. The proposed mechanism consisted of suppressing ICAM-1 and VCAM-1 and a decrease in inflammatory cytokines [68]. These findings are relevant since it was found that miR-125b is upregulated in sepsis and showed a positive correlation with enhanced disease severity, inflammation, and increased mortality in sepsis patients [69]. The previous results further reinforce the role of miR-125 in controlling key steps in the coagulation cascade, through its role in regulating endothelial adhesion proteins, such as ICAM-1 and VCAM-1, and directly regulating the expression of coagulation factors.

Upstream to factor IX in the intrinsic pathway, factor XI can be regulated by miR-181a-5p. It was shown how miR-181a-5p caused a slight, yet significant, decrease in factor XI mRNA levels, thus reducing the protein levels of factor XI [26]. The assays demonstrated a direct interaction between miR-181a-5p and the 3′-UTR of the messenger for factor XI. Importantly, factor XI mRNA levels were inversely and significantly correlated with miR-181a-5p levels, specifically in healthy livers, demonstrating that miR-181a-5p can directly impact the coagulation pathway [70].

In the extrinsic pathway, we can find factor X in the coagulation cascade. It has been found that miR-24 is overexpressed following trauma. However, it negatively correlates with factor X of the coagulation cascade in trauma patients, such that miR-24 is suspected to be able to inhibit factor X synthesis after trauma or trauma-induced coagulopathy [71]. Furthermore, miR-24 can decrease factor X and factor XII mRNA levels through negative regulation of nuclear factor, specifically nuclear factor-4 alpha in liver cells [71]. After performing the corresponding study, the authors concluded that miR-24 overexpression in patients after trauma was involved in the hypocoagulation state by inhibiting factor X and/or factor XII synthesis [71]. Thus, it can be stated that miR-24 plays an anticoagulant role as well as being a suitable biomarker for trauma-induced coagulopathy. MiR-24 could become a new therapeutic treatment for these patients since it has been shown in vitro that pretreatment with miR-24 can significantly suppress factor X levels [71].

Zhang et al. have studied the expression of miR-19a-3p in relation to DIC [72]. Their research revealed the potential of this miRNA as a therapeutic target due to its capability of inhibiting the coagulation cascade by targeting TF. As described above, TF by forming a complex with factor VIIa initiates clotting by activating factor X and factor IX. Both in vitro and in vivo results showed a great improvement in coagulation when miR-19a-3p was administered as a therapy. MiR-19a was able to inhibit the coagulation pathway by directly targeting TF through 3′-UTR binding sites [72]. The PI3K/AKT pathway is directly related to TF expression and coagulation. Factor Xa regulates TF expression in endothelial cells via mitogen-activated protein kinase (MAPK)- and nuclear factor kappa B (NF-κB)-dependent pathways [73,74]. Indirectly, TF regulation by miR-19 was dependent on the NF-κB/IκB pathway and the AKT pathway [72,73]. Therefore, miR-19 seems an interesting axis connecting the coagulation pathway, inflammation pathway, and AKT pathway.

Here it is important to note that recent literature showed how miR-19a was down-regulated while TF was shown to be up-regulated in newborns with sepsis-induced DIC relative to the control group [72]. 

TF expression can also be modulated by miR-223. In this regard, it was demonstrated that miR-223 is one of the most abundant miRNAs in platelets and platelet-derived extracellular vesicles [25,75]. Therefore, this miRNA could be transferred to platelets from monocytic cells and regulate TF expression as it binds to the 3′-UTR of the TF mRNA transcript and thereby inhibits its expression [76,77].

Previously, we have shown how miR-125a can regulate the expression of ICAM-1. Dai et al. demonstrated that miR-223 negatively regulates ICAM-1 expression during an inflammatory process by inhibiting NF-κB [78]. In addition, previous studies showed that miR-223 inhibited inflammation in TNFα-stimulated endothelial cells by down-regulating the MAPK pathway [79]. Furthermore, another study showed that miR-223 levels in endothelial cells were inversely correlated with TF expression in vivo [77].

The before mentioned miRNAs clearly show the impact of miRNA expression in the control of the coagulation process. However, further studies to assess the impact of miRNAs in DIC are needed. In this regard, only an integrative analysis of molecular mechanisms in the context of the control of coagulation factors and endothelial adhesion proteins and mediators that clearly contribute to the coagulation cascade may help to understand the intricate mechanisms underlying sepsis-associated DIC. 

### 2.2. The Role of Long Non-Coding RNAs in DIC

LncRNAs are defined as long transcripts of RNA of more than 200 nucleotides that are not associated with the expression of proteins [80].

Recent studies have shown that sepsis-related cardiac dysfunction can alter the expression of such ncRNAs [81,82]. Moreover, several lncRNAs have been described in sepsis and cardiovascular diseases [81,82]. As such, it is vital that their role becomes established regarding the dysregulation that takes place during DIC. This will in turn permit the description of lncRNA as biomarkers in diagnosis and prognosis, as well as possible therapeutic targets.

Epigenetic changes due to sepsis-related molecular pathways may also influence ncRNAs. Specifically, lncXR_343955 was found to be differentially methylated and may regulate the expression of different CAMs [83]. LINC00341 has also been associated with VCAM expression, inhibiting it [84]. Since these lncRNAs are linked to the expression of CAMs, they indirectly influence the PI3K/AKT pathway [84]. Thus, elucidating their role in the dysregulation of coagulation in DIC may allow the description of different pathological pathways associated with the disease.

The role of lncRNA cancer susceptibility candidate 2 (lncCASC2) has been studied in sepsis by Wang et al. [85]. Its expression was found to be inversely linked to sepsis severity and mortality, proving its use as a potential biomarker [85]. Interestingly, lncCASC2 was also negatively correlated with inflammatory cytokines. As such, its impact on the coagulation pathway, and specifically on endothelial damage, may be worth exploring. LncCASC2 has also been related to inflammation, sponging the inflamma-miR miR-155 [85]. Additionally, the overexpression of lncCASC2 inhibited the NF-κB activation pathway [85]. It is noteworthy that the TF promoter contains binding sites for NF-κB, which entails that the procoagulant activity of TF endothelial cells and subsequent thrombin generation directly depends on the NF-κB [86].

Similarly, lncRNA growth-arrest-specific transcript 5 (lncGAS5) was also associated with the diagnosis and mortality of sepsis [87]. Interestingly, lncGAS5 has also been linked to the NF-κB pathway in sepsis [88]. Moreover, lncGAS5 was also found to impact the PI3K/AKT pathway by targeting miR-223 and miR-21 [89]. 

These studies show the potential role of lncCASC2 and lncGAS5 in indicating the state of the NF-κB pathway and, in turn, endothelial damage. Thus, these lncRNAs should be studied further in the context of DIC to establish their potential as regulatory elements in those pathways regulating coagulation.

LncH19 was one of the first lncRNA described and as such is one of the most extensively studied lncRNAs. Although most research published on this lncRNA is related to cancer due to its oncogenic primary description, a recent study has established lncH19 as a potential prognosis biomarker in sepsis [90]. Additionally, lncH19 showed a mild correlation with cytokines IL-6 and TNF-α, as well as coagulation dysfunction. 

Recently, miR-125a was found to be part of a regulatory axis with lncRNA Metastasis Associated Lung Adenocarcinoma Transcript 1 (lncMALAT1) [91]. In this study, Liu et al. demonstrated the utility of both lncMALAT1 and miR-125a to diagnose sepsis and predict organ injury, inflammation severity, and mortality. In another study, lncMALAT1 was found to promote the progression of acute lung injury related to sepsis, by sponging other inflamma-miRs such as miR-146 and thus indirectly promoting the NF-κB pathway [92] which downstream may affect the PI3K/AKT axis. Furthermore, lncRNA nuclear paraspeckle assembly transcript 1 (lncNEAT1) was also associated with miR-125a [93]. The overexpression of lncNEAT1 was correlated with a greater severity and a higher probability of mortality [93,94].

LncRNAs related to protease-activated receptor 1 (PAR-1) should also be studied due to their effects on the coagulation cascade after secretion from the endothelial cell. One such lncRNA is a non-protein-coding RNA upstream of F2R/PAR1 (ncRuPAR) that has been found to target PAR-1 and reduce indirectly the PI3K/AKT pathway [95]. This lncRNA has also been associated with the expression of VEGF, inhibiting it [96].

Another interesting lncRNA is potassium voltage-gated channel subfamily Q member 1 (KCNQ1) opposite strand/antisense transcript 1 (lncKCNQ1OT1), which has been found to sponge miR-24-3p [97]. This miRNA, as discussed in the previous section, has been studied extensively in both sepsis and DIC. LncKCNQ1OT1 was found to be under-expressed in sepsis patients, while also having a negative correlation to clinical scores related to coagulation [97] Additionally, Luo et al. first determined through in silico analysis, and later proved using experimental procedures, the relationship between lncKCNQ1OT1, miR-24, and vWF [98]. Such an axis should be studied further in the context of DIC since vWF is also a key contributor to the coagulation pathway.

Lastly, some lncRNAs have specifically been associated with factors in the coagulation cascade. Coagulation factor XI antisense RNA 1 (F11-AS1) targets miR-3146 and promotes the expression of phosphatase and tensin homolog (PTEN) [99], which participates in the PI3K/AKT pathway.

### 2.3. The Role of Circular RNAs in DIC

One of the most important characteristics of circRNAs is that these circular nucleic acids are present in most mammalian tissues and are highly expressed in specific tissues, being also present in plasma [34,100]. Moreover, circRNAs have shown differential expression levels at distinct development stages [101,102]. Due to their potential role in regulating transcriptional programs, circRNAs have been proposed as potential biomarkers for several diseases [38], including sepsis, as we recently reviewed [103]. Interestingly, several circRNAs have been described to function as miRNA “sponges”, reducing the availability of such miRNAs. This in turn causes modulation of gene expression, transcription, and protein translation [41], so circRNAs are feasible key modulatory factors and mediators participating in mechanisms underlying pathological processes such as DIC.

Despite the advances in this field, it is noteworthy that further biological contextualization of these circRNAs by exploring gene ontology (GO) and Kyoto Encyclopedia of Genes and Genomes (KEGG) pathways may contribute to demonstrating that these circRNAs are key mediators in inflammatory response (e.g., cytosine receptor interaction, TNF-α signaling pathway, NF-κB signaling pathway, and chemokine signaling pathway), leukocyte rolling and adhesion on endothelial cells, platelet function (activation, aggregation, and adhesion), and DIC. Moreover, even though the potential of circRNA biomarkers has been established [104], they are mainly studied and evaluated in the context of cancer, with little research focusing on the role of circRNA in sepsis and DIC. Though we will focus on established relationships directly with sepsis, some connections provided by cancer studies may be useful to find relations between described circRNAs and their potential regulation of miRNAs and molecular pathways related to sepsis and DIC. 

In sepsis, some circRNAs have been postulated as promising biomarkers. This is the case of circRNA guanine nucleotide exchange factor for Ras-like small GTPases (RasGEF) domain family member 1B (circRasGEF1B), an LPS-inducible circRNA in animal models of sepsis, which modulates the stability of *ICAM-1* mRNA [105]. It was observed by Nie et al. that LPS stimulation leads to the activation of endothelial cells, promoting the release of inflammatory mediators (e.g., IL-1β, IL-18, TNF-α, chemokines, monocyte chemoattractant protein-1 (MCP-1), etc.), which further promote vascular endothelial damage [32]. The study of Nie et al. proposes that during sepsis, circRNAs can modulate vascular function by influencing the increment of nitric oxide levels and by down-regulating the antioxidant defense system [32]. Besides the role of circRasGEF1B in regulating ICAM-1 levels, both circ_0007456 and circRasGEF1B have been directly linked to the expression of ICAM-1 [105,106]. Closely related to the expression of ICAM-1 is the VCAM-1, both being downstream proteins of the PI3K/AKT pathway. Several studies have indicated that circRNA nephrocystin 4 (circNPHP4) expression has a significant impact on the expression of both molecules, specifically by sponging miR-1231 [107]. In addition, VCAM-1 also interacts with E-selectin and Lai et al. have found a relation between the expression of circ_0079662, miR-324, and HOXA9 [108].

CircRNA fatty acid desaturase 2 (circFADS2) is an important circRNA that has been found in low levels in septic patients [109]. CircFADS2 counteracts the function of miR-133a and miR-122a. Surprisingly, although circFADS2 blocks the function of miR-133a, this miRNA is usually increased in severe septic patients with unfavorable prognoses [110]. Furthermore, circFADS2 can suppress cell apoptosis by down-regulating miR-122a through the interaction with the miR-498/mammalian target of rapamycin (mTOR) axis to protect chondrocytes from apoptosis induced by LPS and inflammation [110]. This is quite exciting because it was described how mTOR is required for Bcl-3 activation, which in turn participates in the condensation of fibrin by activated platelets [111].

The role of AKT/mTOR axis in platelet activity and thrombosis is being uncovered [112]. Particularly, abnormal autophagic death of endothelial cells has been shown to affect plaque and promote thrombosis, which is related to this axis; thus it is feasible that circ_0030042 could play an essential role in vascular function [113].

Also related to the AKT/mTOR axis, Zeng et al. demonstrated that the overexpression of circRNA angiomotin like 1 (circAmotl1) protects against cardiomyopathy by binding to 3-phosphoinositide-dependent kinase 1 (PDK1) and AKT1 in vivo [114]. 

The AKT/mTOR axis is also directly involved in controlling the expression of TF to initiate thrombosis [115]. Interestingly, as described above, miR-19a-3p targets TF so it has been directly related to DIC. Thus, elucidating the possible circRNAs that may regulate this miRNA can be useful in the design of therapeutic approaches. CircRNA hippocampus abundant transcript 1 (circHIAT1) or circ_0000096 has been proven to modulate the expression of miR-19a-3p in cervical cancer [116]. The decrease in circHIAT1 increases miR-19a-3p expression which in turn produces the increase in AKT and mTOR. Therefore, the ability of circHIAT1 to sponge miR-19a-3p may directly affect the pathway. Another study carried out by Lu et al. related, in type 2 diabetes, the increase in miR-19a-3p to a decrease in circ_0063425 and the PI3K/AKT pathway [117].

These studies show that circ_0000096 and circ_0063425 regulate the expression of miR-19a-3p resulting in a modulation of the PI3K/AKT/mTOR pathway. These signaling molecules have been shown to regulate the expression of TF [118]. Thus, the aforementioned circRNAs should be studied further in the context of the coagulation cascade to determine their possible involvement in DIC-related dysfunction. This idea of the important role of circRNAs in DIC acquires relevance because there is evidence of the important role of the PI3K/AKT pathway in the suppression of LPS-induced inflammation and coagulation in the endotoxemic mice model [74]. Downstream to the PI3K/AKT/mTOR pathway, AKT targets Forkhead box O (FOXO) transcription factors [119]. In this pathway, Yuan et al. predicted through in silico analysis that hsa_circ_0039466 sponges miR-96 in hepatocellular carcinoma which impacts FOXO1 expression [120]; thus hsa_circ_0039466 may also indirectly regulate TF, as we have previously described for hsa_circ_0000096 and hsa_circ_0063425. Additionally, circRNA PTEN (circPTEN) or circ_0094342 modulates the inflamma-miR miR-155 and miR-330, regulating the expression of PTEN/PI3K/AKT [121]. 

As described above, miR-122-5p has been related to coagulation malfunctioning in sepsis, and altered levels of this miRNA have been found in patients with altered coagulation parameters (i.e., aPTT, fibrinogen, and antithrombin) [67]. Therefore, it is necessary to explore the possible expression dysfunction of the circRNA sponges.

Hsa_circ_0005963 or ciRS-122 sponges miR-122, decreasing its expression and increasing expression of PKM2 [122], like hsa_circ_000826 which sponges miR-330 [123]. CircPTN has also been found to sponge miR-122 and, in turn, modulate the expression of SOX6 [124].

Notably, some studies have focused on the relation between miR-122 and the cytoplasmic polyadenylation element-binding protein 1 (CPEB1)/PTEN/AMPK/mTOR axis, which we have shown is a relevant metabolic axis related to coagulation. Specifically, circRNA_002581 has been found to directly influence the expression of CPEB1 [125,126] which, besides controlling this axis, has been found to regulate the pathological expression of VEGF [127]. Another relevant circRNA related to VEGF is circRNA fibronectin type III domain containing 3B (circFndc3b) [128]. CircFndc3b has been found in cardiac endothelial cells to be contributing to the overexpression of VEGF which in turn increases its angiogenic activity in endothelial cells [128]. As is widely known, VEGF is involved in the hemostasis of endothelial cells by participating in several processes such as vascular permeability, VEGF stimulates endothelial cell proliferation, migration, and tube formation [129]. Recent studies suggest that VEGF is involved in the thrombotic process by stimulating the expression of TF in vascular endothelial cells [130]. Interestingly, TF can also stimulate the transcription of the gene encoding VEGF through a positive feedback process [131]. Importantly, VEGF, via the formation of the TF–factor VIIa complex, can trigger the extrinsic coagulation cascade [132,133,134]. In addition, VEGF can also increase the expression of thrombomodulin (TM) in endothelial cells. When complexed with thrombin in the membrane of endothelial cells this increases levels of APC, which is an inhibitor of the coagulation cascade, through the neutralization of activated factor V and activated factor VIII. Moreover, VEGF also increases the activation of thrombin-activatable fibrinolysis inhibitor (TAFI) by >1000-fold, which is involved in the inhibition of clot lysis [135].

Therefore, the increase in knowledge of the control of VEGF by circFndc3b may result in further interest in the comprehension of the epigenetic regulation of the coagulation process, mainly in those syndromes such as DIC.

Lastly, several circRNA have been linked to vWF, such as hsa_circ_0025119 [136], hsa_circ_0000698, hsa_circ_0002775, hsa_circ_0005585, and hsa_circ_0043837 [137]. These last circRNAs have also been found to sponge miR-16, which directly impacts vWF expression.

After reviewing the recent advances, we can identify common pathways that are likely altered and regulated by specific ncRNAs. Most notably, the PI3K/AKT pathway and associated proteins and pathways are altered after NF-κB activation and by the effect of TF. Several circRNAs have been associated with the dysregulation of these pathways, such as circHIAT1, circFADS2, circ_0000096, and circ_0063425. Moreover, the expression of VEGF is altered and is likely modulated by diverse circRNAs including circ_002581 and circFndc3b.

## 3. Discussion and Future Perspectives

Recent research in epigenetics, particularly in ncRNAs, will contribute to understanding molecular processes featuring the different phenotypes found in sepsis. One of the most severe processes occurring in sepsis is DIC.

Current concerns on sepsis and sepsis-related pathologies lie in the diagnostic tools since they should be rapid, feasible, and reproducible. As such, biological and clinical parameters are not sufficient and do not meet the healthcare demands, with an increasing number of sepsis patients each year. Particularly for DIC assessment, the International Society on Thrombosis and Haemostasis (ISTH) DIC score can be used, but this score has shown a moderate performance in identifying overt DIC [138]. Moreover, this score remains to be investigated in contemporary patient populations and it has not been demonstrated to be enough to anticipate clinical decisions, so the use of biomarkers seems to be a current need in early DIC identification. 

NcRNAs have been associated with coagulation hemostasis dysregulation in several diseases and have helped establish the implicated and affected pathways in these disorders. Specifically, in DIC, some studies have identified certain axes involved. However, a more comprehensive intertwined web must be established to observe the entire impact DIC has, particularly at the cellular and molecular levels. In this regard, recent molecular research supported by the characterization of epigenetic signatures based on ncRNAs and subsequent exploration of GO and KEGG pathways may contribute to demonstrating that these ncRNAs are key mediators in the inflammatory response (e.g., cytosine receptor interaction, TNF-α signaling pathway, NF-κB signaling pathway, and chemokine signaling pathway), leukocyte rolling and adhesion on endothelial cells, platelet function (activation, aggregation, and adhesion), and the PI3K/AKT/mTOR axis to consequently lead to DIC.

Since ncRNAs are critical novel regulators of all these molecular pathways, they are important candidates to improve diagnostics and prognosis and set the basis to design novel therapeutic approaches for DIC. 

**Figure 3:**
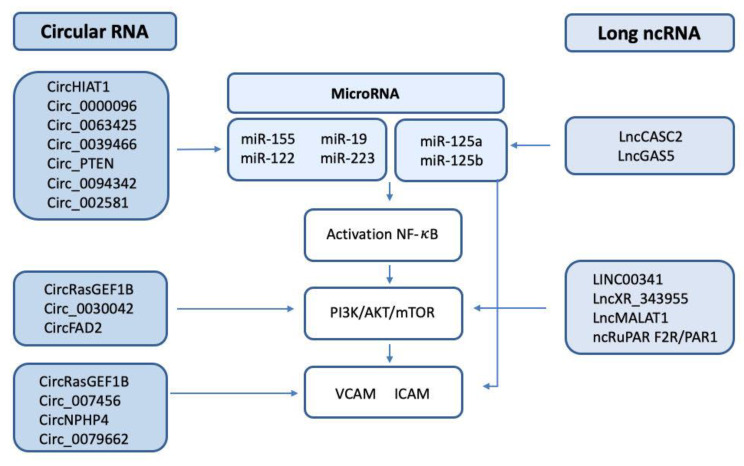
Scheme of the circRNA/lncRNA/miRNA involved in the regulation of NF-κB, the PI3K/AKT/mTOR pathway, and the endothelial adhesion factors VCAM and ICAM.

As we described in this review, several ncRNAs (lncRNAs, miRNAs, and circRNAs) can directly control the expression and function of several factors participating throughout the coagulation cascade, leading to DIC in sepsis. Moreover, intricate interactions have been found among miRNAs, circRNAs, and lncRNAs (Figure 3), which can affect the control of the PI3K/AKT/mTOR pathway, which is directly related to vascular endothelium activation [139], coagulation [74], platelet activity, and thrombosis [112].

Among the ncRNAs described in this work, miR-122-5p has been directly associated with coagulation abnormalities in septic patients [67]. Intriguingly, circ_0005963 or ciRS-122 [122] and circPTN [124], which sponge miR-122, have also been related to coagulation, thus adding additional points of control to this mechanism. Notably, as described in this manuscript, the impact of miR-122 on the CPEB1/PTEN/AMPK/mTOR axis makes the expression of this miRNA a relevant switch in coagulation.

Zhang et al. revealed the potential of miR-19a-3p to inhibit the coagulation cascade by targeting TF, directly linking this miRNA with DIC [77]. Importantly, circHIAT1 or circ_0000096 [116] and circ_0063425 [117] have shown their ability to control the expression of miR-19a-3p, therefore directly resulting in the modulation of the PI3K/AKT/mTOR pathway and affecting the expression of key factors such as TF, VCAM-1, and ICAM-1, among others.

Using in vivo studies, it has been found that miR-223 in endothelial cells was inversely correlated with TF expression [77]. In this regard, lncGAS5 targets miR-223, adding a new additional step of control of the PI3K/AKT pathway [89] and providing additional relevance to the PI3K/AKT axis in the control of coagulation. In this line, lncRuPAR can inhibit the PI3K/AKT pathway [95], indirectly affecting the coagulation cascade [96]. LncKCNQ1OT1 has been found to sponge miR-24-3p [97], an event which may be relevant in DIC since miR-24 overexpression participates in the hypocoagulation state by inhibiting factor X and/or factor XII synthesis [71]. 

In the coagulation cascade, the expression of endothelial adhesion factors is also a key process with a direct impact on the formation of the clot. Particularly, ICAM-1 and VCAM-1 proteins, which are downstream of the NF-κB/PI3K/AKT pathway, can be regulated by lncRNAs such as lncXR_343955 [83] and circNPHP4 [107]. In addition, VCAM-1 can also be inhibited by LINC00341 [84] and interacts with circ_0079662, miR-324, and HOXA9 [108].

As discussed, the expression of ncRNAs affects the intricate network of interactions among factors in the coagulation cascade, endothelial adhesion factors, and other mediators. Furthermore, lncRNAs and circRNAs can sponge miRNAs which directly regulate most of these factors participating in the coagulation cascade and the formation of the clot. As we have deduced, further research needs to be carried out on potential new biomarkers based on these ncRNAs, which could also help us to understand the underlying molecular mechanisms taking place. Continuous investigation in this field is helping to elucidate the potential altered pathways in DIC, so this research will help in providing new potential biomarkers, and accelerate the application of therapies that will ultimately lead to an increase in patient survival.

## Data Availability

Not applicable.

## References

[1] Singer M., Deutschman C.S., Seymour C.W., Shankar-Hari M., Annane D., Bauer M., Bellomo R., Bernard G.R., Chiche J.-D., Coopersmith C.M. (2016). The Third International Consensus Definitions for Sepsis and Septic Shock (Sepsis-3). JAMA.

[2] Rudd K.E., Johnson S.C., Agesa K.M., Shackelford K.A., Tsoi D., Kievlan D.R., Colombara D.V., Ikuta K.S., Kissoon N., Finfer S. (2020). Global, Regional, and National Sepsis Incidence and Mortality, 1990–2017: Analysis for the Global Burden of Disease Study. Lancet.

[3] Ince C., Mayeux P.R., Nguyen T., Gomez H., Kellum J.A., Ospina-Tascón G.A., Hernandez G., Murray P., De Backer D. (2016). The Endothelium In Sepsis. Shock.

[4] Bermejo-Martin J.F., Martín-Fernandez M., López-Mestanza C., Duque P., Almansa R. (2018). Shared Features of Endothelial Dysfunction between Sepsis and Its Preceding Risk Factors (Aging and Chronic Disease). J. Clin. Med..

[5] Reinhart K., Bayer O., Brunkhorst F., Meisner M. (2002). Markers of Endothelial Damage in Organ Dysfunction and Sepsis. Crit. Care Med..

[6] Semeraro N., Colucci M., Ten Cate H., Levi M. (2003). Endothelial Cell Perturbation and Disseminated Intravascular Coagulation. Molecular Mechanisms of Disseminated Intravascular Coagulation.

[7] Semeraro N., Ammollo C.T., Semeraro F., Colucci M. (2012). Sepsis, Thrombosis and Organ Dysfunction. Thromb. Res..

[8] Taylor F., Toh C.-H., Hoots K., Wada H., Levi M. (2001). Towards Definition, Clinical and Laboratory Criteria, and a Scoring System for Disseminated Intravascular Coagulation: On Behalf of the Scientific Subcommittee on Disseminated Intravascular Coagulation (DIC) of the International Society on Thrombosis and Haemostasis (ISTH). Thromb. Haemost..

[9] Gando S., Levi M., Toh C.-H. (2016). Disseminated Intravascular Coagulation. Nat. Rev. Dis. Primer.

[10] Ikezoe T. (2015). Thrombomodulin/Activated Protein C System in Septic Disseminated Intravascular Coagulation. J. Intensive Care.

[11] Gando S., Saitoh D., Ogura H., Mayumi T., Koseki K., Ikeda T., Ishikura H., Iba T., Ueyama M., Eguchi Y. (2008). Natural History of Disseminated Intravascular Coagulation Diagnosed Based on the Newly Established Diagnostic Criteria for Critically Ill Patients: Results of a Multicenter, Prospective Survey. Crit. Care Med..

[12] Vincent J.-L., Castro P., Hunt B.J., Jörres A., Praga M., Rojas-Suarez J., Watanabe E. (2018). Thrombocytopenia in the ICU: Disseminated Intravascular Coagulation and Thrombotic Microangiopathies—What Intensivists Need to Know. Crit. Care.

[13] Oto J., Fernández-Pardo Á., Miralles M., Plana E., España F., Navarro S., Medina P. (2020). Activated Protein C Assays: A Review. Clin. Chim. Acta.

[14] Wada H., Matsumoto T., Yamashita Y., Hatada T. (2014). Disseminated Intravascular Coagulation: Testing and Diagnosis. Clin. Chim. Acta.

[15] Kuenen B.C., Levi M., Meijers J.C.M., Kakkar A.K., van Hinsbergh V.W.M., Kostense P.J., Pinedo H.M., Hoekman K. (2002). Analysis of Coagulation Cascade and Endothelial Cell Activation During Inhibition of Vascular Endothelial Growth Factor/Vascular Endothelial Growth Factor Receptor Pathway in Cancer Patients. Arterioscler. Thromb. Vasc. Biol..

[16] Pepper M.S., Ferrara N., Orci L., Montesano R. (1991). Vascular Endothelial Growth Factor (VEGF) Induces Plasminogen Activators and Plasminogen Activator Inhibitor-1 in Microvascular Endothelial Cells. Biochem. Biophys. Res. Commun..

[17] Mandriota S.J., Pepper M.S. (1997). Vascular Endothelial Growth Factor-Induced in Vitro Angiogenesis and Plasminogen Activator Expression Are Dependent on Endogenous Basic Fibroblast Growth Factor. J. Cell Sci..

[18] Kroon M.E., Koolwijk P., Vermeer M.A., van der Vecht B., van Hinsbergh V.W.M. (2001). Vascular Endothelial Growth Factor Enhances the Expression of Urokinase Receptor in Human Endothelial Cells via Protein Kinase C Activation. Thromb. Haemost..

[19] Chavda V., Madhwani K. (2021). Coding and Non-Coding Nucleotides’: The Future of Stroke Gene Therapeutics. Genomics.

[20] Liu N., Wang Z.-Z., Zhao M., Zhang Y., Chen N.-H. (2020). Role of Non-Coding RNA in the Pathogenesis of Depression. Gene.

[21] Zhang X., Hamblin M.H., Yin K.-J. (2019). Noncoding RNAs and Stroke. Neuroscientist.

[22] Faraldi M., Gomarasca M., Banfi G., Lombardi G. (2018). Free Circulating MiRNAs Measurement in Clinical Settings. Adv. Clin. Chem..

[23] O’Brien J., Hayder H., Zayed Y., Peng C. (2018). Overview of MicroRNA Biogenesis, Mechanisms of Actions, and Circulation. Front. Endocrinol..

[24] He L., Hannon G.J. (2004). MicroRNAs: Small RNAs with a Big Role in Gene Regulation. Nat. Rev. Genet..

[25] Willeit P., Zampetaki A., Dudek K., Kaudewitz D., King A., Kirkby N.S., Crosby-Nwaobi R., Prokopi M., Drozdov I., Langley S.R. (2013). Circulating MicroRNAs as Novel Biomarkers for Platelet Activation. Circ. Res..

[26] Salloum-Asfar S., Teruel-Montoya R., Arroyo A.B., García-Barberá N., Chaudhry A., Schuetz E., Luengo-Gil G., Vicente V., González-Conejero R., Martínez C. (2014). Regulation of Coagulation Factor XI Expression by MicroRNAs in the Human Liver. PLoS ONE.

[27] Morelli V.M., Brækkan S.K., Hansen J.-B. (2020). Role of MicroRNAs in Venous Thromboembolism. Int. J. Mol. Sci..

[28] Fort A., Borel C., Migliavacca E., Antonarakis S.E., Fish R.J., Neerman-Arbez M. (2010). Regulation of Fibrinogen Production by MicroRNAs. Blood.

[29] Teruel R., Pérez-Sánchez C., Corral J., Herranz M.T., Pérez-Andreu V., Saiz E., García-Barberá N., Martínez-Martínez I., Roldán V., Vicente V. (2011). Identification of MiRNAs as Potential Modulators of Tissue Factor Expression in Patients with Systemic Lupus Erythematosus and Antiphospholipid Syndrome. J. Thromb. Haemost..

[30] Bhatti G.K., Khullar N., Sidhu I.S., Navik U.S., Reddy A.P., Reddy P.H., Bhatti J.S. (2021). Emerging Role of Non-coding RNA in Health and Disease. Metab. Brain Dis..

[31] Morillon A., Morillon A. (2018). 2-Definition and Families of Long Non-Coding RNA. Long Non-coding RNA.

[32] Nie M.-W., Han Y.-C., Shen Z.-J., Xie H.-Z. (2020). Identification of CircRNA and MRNA Expression Profiles and Functional Networks of Vascular Tissue in Lipopolysaccharide-Induced Sepsis. J. Cell. Mol. Med..

[33] Yu Z., Huang Q., Zhang Q., Wu H., Zhong Z. (2021). CircRNAs Open a New Era in the Study of Cardiovascular Disease (Review). Int. J. Mol. Med..

[34] Rybak-Wolf A., Stottmeister C., Glažar P., Jens M., Pino N., Giusti S., Hanan M., Behm M., Bartok O., Ashwal-Fluss R. (2015). Circular RNAs in the Mammalian Brain Are Highly Abundant, Conserved, and Dynamically Expressed. Mol. Cell.

[35] Huang S., Yang B., Chen B.J., Bliim N., Ueberham U., Arendt T., Janitz M. (2017). The Emerging Role of Circular RNAs in Transcriptome Regulation. Genomics.

[36] Bolha L., Ravnik-Glavač M., Glavač D. (2017). Circular RNAs: Biogenesis, Function, and a Role as Possible Cancer Biomarkers. Int. J. Genom..

[37] Dragomir M., Calin G.A. (2018). Circular RNAs in Cancer-Lessons Learned From MicroRNAs. Front. Oncol..

[38] Beltrán-García J., Osca-Verdegal R., Nacher-Sendra E., Pallardó F.V., García-Giménez J.L. (2020). Circular RNAs in Sepsis: Biogenesis, Function, and Clinical Significance. Cells.

[39] Hashemian S.M., Pourhanifeh M.H., Fadaei S., Velayati A.A., Mirzaei H., Hamblin M.R. (2020). Non-Coding RNAs and Exosomes: Their Role in the Pathogenesis of Sepsis. Mol. Ther. Nucleic Acids.

[40] Panda A.C., Xiao J. (2018). Circular RNAs Act as MiRNA Sponges. Circular RNAs: Biogenesis and Functions.

[41] Huang A., Zheng H., Wu Z., Chen M., Huang Y. (2020). Circular RNA-Protein Interactions: Functions, Mechanisms, and Identification. Theranostics.

[42] Memtsas V.P., Arachchillage D.R.J., Gorog D.A. (2021). Role, Laboratory Assessment and Clinical Relevance of Fibrin, Factor XIII and Endogenous Fibrinolysis in Arterial and Venous Thrombosis. Int. J. Mol. Sci..

[43] Song J.W., Choi J.R., Song K.S., Rhee J.H. (2006). Plasma Factor XIII Activity in Patients with Disseminated Intravascular Coagulation. Yonsei Med. J..

[44] Gailani D., Broze G.J. (1991). Factor XI Activation in a Revised Model of Blood Coagulation. Science.

[45] Lippi G., Favaloro E.J., Franchini M., Guidi G.C. (2009). Milestones and Perspectives in Coagulation and Hemostasis. Semin. Thromb. Hemost..

[46] Levi M., Van Der Poll T., Ten Cate H., Van Deventer S.J.H. (1997). The Cytokine-Mediated Imbalance between Coagulant and Anticoagulant Mechanisms in Sepsis and Endotoxaemia. Eur. J. Clin. Investig..

[47] Nightingale T., Cutler D. (2013). The Secretion of von Willebrand Factor from Endothelial Cells; an Increasingly Complicated Story. J. Thromb. Haemost..

[48] Federici A.B. (2003). The Factor VIII/von Willebrand Factor Complex: Basic and Clinical Issues. Haematologica.

[49] Lam F.W., Cruz M.A., Leung H.-C.E., Parikh K.S., Smith C.W., Rumbaut R.E. (2013). Histone Induced Platelet Aggregation Is Inhibited by Normal Albumin. Thromb. Res..

[50] Joffre J., Hellman J., Ince C., Ait-Oufella H. (2020). Endothelial Responses in Sepsis. Am. J. Respir. Crit. Care Med..

[51] Dolmatova E.V., Wang K., Mandavilli R., Griendling K.K. (2020). The Effects of Sepsis on Endothelium and Clinical Implications. Cardiovasc. Res..

[52] Sano H., Nakagawa N., Nakajima H., Yoshida S., Iwamoto I. (1995). Role of Vascular Cell Adhesion Molecule-1 and Platelet-Activating Factor in Selective Eosinophil Migration across Vascular Endothelial Cells. Int. Arch. Allergy Immunol..

[53] Hack C.E. (2000). Tissue Factor Pathway of Coagulation in Sepsis. Crit. Care Med..

[54] Sharma S., Tyagi T., Antoniak S. (2022). Platelet in Thrombo-Inflammation: Unraveling New Therapeutic Targets. Front. Immunol..

[55] Mammen E.F. (1998). Antithrombin: Its Physiological Importance and Role in DIC. Semin. Thromb. Hemost..

[56] Morello F., Perino A., Hirsch E. (2009). Phosphoinositide 3-Kinase Signalling in the Vascular System. Cardiovasc. Res..

[57] Puri K.D., Doggett T.A., Huang C.-Y., Douangpanya J., Hayflick J.S., Turner M., Penninger J., Diacovo T.G. (2005). The Role of Endothelial PI3Kγ Activity in Neutrophil Trafficking. Blood.

[58] Puri K.D., Doggett T.A., Douangpanya J., Hou Y., Tino W.T., Wilson T., Graf T., Clayton E., Turner M., Hayflick J.S. (2004). Mechanisms and Implications of Phosphoinositide 3-Kinase δ in Promoting Neutrophil Trafficking into Inflamed Tissue. Blood.

[59] Lazaar A.L., Krymskaya V.P., Das S.K.P. (2001). VCAM-1 Activates Phosphatidylinositol 3-Kinase and Induces P120Cbl Phosphorylation in Human Airway Smooth Muscle Cells 1. J. Immunol..

[60] Tsoyi K., Jang H.J., Nizamutdinova I.T., Park K., Kim Y.M., Kim H.J., Seo H.G., Lee J.H., Chang K.C. (2010). PTEN Differentially Regulates Expressions of ICAM-1 and VCAM-1 through PI3K/Akt/GSK-3β/GATA-6 Signaling Pathways in TNF-α-Activated Human Endothelial Cells. Atherosclerosis.

[61] Shah M.Y., Calin G.A. (2013). The Mix of Two Worlds: Non-Coding RNAs and Hormones. Nucleic Acid Ther..

[62] Wang H., Bei Y., Huang P., Zhou Q., Shi J., Sun Q., Zhong J., Li X., Kong X., Xiao J. (2016). Inhibition of MiR-155 Protects Against LPS-Induced Cardiac Dysfunction and Apoptosis in Mice. Mol. Ther. Nucleic Acids.

[63] Etzrodt V., Idowu T.O., Schenk H., Seeliger B., Prasse A., Thamm K., Pape T., Müller-Deile J., van Meurs M., Thum T. (2021). Role of Endothelial MicroRNA 155 on Capillary Leakage in Systemic Inflammation. Crit. Care.

[64] Ntanyane Phasha M.-A., Soma P., Van Rooy M., Phulukdaree A. (2023). MicroRNA 155, Factor XIII and Type 2 Diabetes Mellitus and Coronary Heart Disease. Curr. Diabetes Rev..

[65] Wang H., Zhang P., Chen W., Feng D., Jia Y., Xie L. (2012). Serum MicroRNA Signatures Identified by Solexa Sequencing Predict Sepsis Patients’ Mortality: A Prospective Observational Study. PLoS ONE.

[66] Wang H., Zhang P., Chen W., Feng D., Jia Y., Xie L. (2012). Four Serum MicroRNAs Identified as Diagnostic Biomarkers of Sepsis. J. Trauma Acute Care Surg..

[67] Wang H.-J., Deng J., Wang J.-Y., Zhang P.-J., Xin Z., Xiao K., Feng D., Jia Y.-H., Liu Y.-N., Xie L.-X. (2014). Serum MiR-122 Levels Are Related to Coagulation Disorders in Sepsis Patients. Clin. Chem. Lab. Med. CCLM.

[68] Ma H., Wang X., Ha T., Gao M., Liu L., Wang R., Yu K., Kalbfleisch J.H., Kao R.L., Williams D.L. (2016). MicroRNA-125b Prevents Cardiac Dysfunction in Polymicrobial Sepsis by Targeting TRAF6-Mediated Nuclear Factor ΚB Activation and P53-Mediated Apoptotic Signaling. J. Infect. Dis..

[69] Zhu X. (2019). MiR-125b but Not MiR-125a Is Upregulated and Exhibits a Trend to Correlate with Enhanced Disease Severity, Inflammation, and Increased Mortality in Sepsis Patients. J. Clin. Lab. Anal..

[70] Nourse J., Danckwardt S. (2021). A Novel Rationale for Targeting FXI: Insights from the Hemostatic MicroRNA Targetome for Emerging Anticoagulant Strategies. Pharmacol. Ther..

[71] Chen L.-J., Yang L., Cheng X., Xue Y.-K., Chen L.-B. (2017). Overexpression of MiR-24 Is Involved in the Formation of Hypocoagulation State after Severe Trauma by Inhibiting the Synthesis of Coagulation Factor X. Dis. Markers.

[72] Zhang R., Lu S., Yang X., Li M., Jia H., Liao J., Jing Q., Wu Y., Wang H., Xiao F. (2021). MiR-19a-3p Downregulates Tissue Factor and Functions as a Potential Therapeutic Target for Sepsis-Induced Disseminated Intravascular Coagulation. Biochem. Pharmacol..

[73] Jiang R., Wang N.-P., Tanaka K.A., Levy J.H., Guyton R.A., Zhao Z.-Q., Vinten-Johansen J. (2011). Factor Xa Induces Tissue Factor Expression in Endothelial Cells by P44/42 MAPK and NF-ΚB-Dependent Pathways. J. Surg. Res..

[74] Schabbauer G., Tencati M., Pedersen B., Pawlinski R., Mackman N. (2004). PI3K-Akt Pathway Suppresses Coagulation and Inflammation in Endotoxemic Mice. Arterioscler. Thromb. Vasc. Biol..

[75] Laffont B., Corduan A., Plé H., Duchez A.-C., Cloutier N., Boilard E., Provost P. (2013). Activated Platelets Can Deliver MRNA Regulatory Ago2•microRNA Complexes to Endothelial Cells via Microparticles. Blood.

[76] Li S., Chen H., Ren J., Geng Q., Song J., Lee C., Cao C., Zhang J., Xu N. (2014). MicroRNA-223 Inhibits Tissue Factor Expression in Vascular Endothelial Cells. Atherosclerosis.

[77] Zhang X., Yu H., Lou J.R., Zheng J., Zhu H., Popescu N.-I., Lupu F., Lind S.E., Ding W.-Q. (2011). MicroRNA-19 (MiR-19) Regulates Tissue Factor Expression in Breast Cancer Cells. J. Biol. Chem..

[78] Li J., Tan M., Xiang Q., Zhou Z., Yan H. (2017). Thrombin-Activated Platelet-Derived Exosomes Regulate Endothelial Cell Expression of ICAM-1 via MicroRNA-223 during the Thrombosis-Inflammation Response. Thromb. Res..

[79] Dai G.-H., Ma P.-Z., Song X.-B., Liu N., Zhang T., Wu B. (2014). MicroRNA-223-3p Inhibits the Angiogenesis of Ischemic Cardiac Microvascular Endothelial Cells via Affecting RPS6KB1/Hif-1a Signal Pathway. PLoS ONE.

[80] Bridges M.C., Daulagala A.C., Kourtidis A. (2021). LNCcation: LncRNA Localization and Function. J. Cell Biol..

[81] Wang C., Liang G., Shen J., Kong H., Wu D., Huang J., Li X. (2021). Long Non-Coding RNAs as Biomarkers and Therapeutic Targets in Sepsis. Front. Immunol..

[82] Li J., Zhang Y., Zhang D., Li Y. (2021). The Role of Long Non-Coding RNAs in Sepsis-Induced Cardiac Dysfunction. Front. Cardiovasc. Med..

[83] Shen Z.-J., Han Y.-C., Nie M.-W., Wang Y.-N., Xiang R.-L., Xie H.-Z. (2021). Genome-Wide Identification of Altered RNA M6A Profiles in Vascular Tissue of Septic Rats. Aging.

[84] Huang T.-S., Wang K.-C., Quon S., Nguyen P., Chang T.-Y., Chen Z., Li Y.-S., Subramaniam S., Shyy J., Chien S. (2017). LINC00341 Exerts an Anti-Inflammatory Effect on Endothelial Cells by Repressing VCAM1. Physiol. Genom..

[85] Wang R., Zhao J., Wei Q., Wang H., Zhao C., Hu C., Han Y., Hui Z., Yang L., Dai Q. (2022). Potential of Circulating LncRNA CASC2 as a Biomarker in Reflecting the Inflammatory Cytokines, Multi-organ Dysfunction, Disease Severity, and Mortality in Sepsis Patients. J. Clin. Lab. Anal..

[86] Yang X., Li L., Liu J., Lv B., Chen F. (2016). Extracellular Histones Induce Tissue Factor Expression in Vascular Endothelial Cells via TLR and Activation of NF-ΚB and AP-1. Thromb. Res..

[87] Zhang W., Chen B., Chen W. (2022). LncRNA GAS5 Relates to Th17 Cells and Serves as a Potential Biomarker for Sepsis Inflammation, Organ Dysfunctions and Mortality Risk. J. Clin. Lab. Anal..

[88] Gao H., Ma H., Gao M., Chen A., Zha S., Yan J. (2021). Long Non-Coding RNA GAS5 Aggravates Myocardial Depression in Mice with Sepsis via the MicroRNA-449b/HMGB1 Axis and the NF-ΚB Signaling Pathway. Biosci. Rep..

[89] Zheng D., Hou Y., Li Y., Bian Y., Khan M., Li F., Huang L., Qiao C. (2020). Long Non-Coding RNA Gas5 Is Associated With Preeclampsia and Regulates Biological Behaviors of Trophoblast via MicroRNA-21. Front. Genet..

[90] Yu B., Cui R., Lan Y., Zhang J., Liu B. (2021). Long Non-Coding RNA H19 as a Diagnostic Marker in Peripheral Blood of Patients with Sepsis. Am. J. Transl. Res..

[91] Liu W., Geng F., Yu L. (2020). Long Non-coding RNA MALAT1/MicroRNA 125a Axis Presents Excellent Value in Discriminating Sepsis Patients and Exhibits Positive Association with General Disease Severity, Organ Injury, Inflammation Level, and Mortality in Sepsis Patients. J. Clin. Lab. Anal..

[92] Liang W.-J., Zeng X.-Y., Jiang S.-L., Tan H.-Y., Yan M.-Y., Yang H.-Z. (2020). Long Non-Coding RNA MALAT1 Sponges MiR-149 to Promote Inflammatory Responses of LPS-Induced Acute Lung Injury by Targeting MyD88. Cell Biol. Int..

[93] Yang Y., Yang L., Liu Z., Wang Y., Yang J. (2020). Long Noncoding RNA NEAT 1 and Its Target MicroRNA-125a in Sepsis: Correlation with Acute Respiratory Distress Syndrome Risk, Biochemical Indexes, Disease Severity, and 28-day Mortality. J. Clin. Lab. Anal..

[94] Huang Q., Huang C., Luo Y., He F., Zhang R. (2018). Circulating LncRNA NEAT1 Correlates with Increased Risk, Elevated Severity and Unfavorable Prognosis in Sepsis Patients. Am. J. Emerg. Med..

[95] Xu R., Yu J., Song S., Sun D., Xiu L., Xu J., Zhao J., Liu X., Ji Q., Yue X. (2022). Long Non-Coding RNA NcRuPAR Regulates Gastric Cancer Cell Proliferation and Apoptosis via Phosphoinositide 3-Kinase/Protein Kinase B Signaling. Int. J. Med. Sci..

[96] Liu L., Yan B., Yang Z., Zhang X., Gu Q., Yue X. (2014). NcRuPAR Inhibits Gastric Cancer Progression by Down-Regulating Protease-Activated Receptor-1. Tumor Biol..

[97] Jiao W., Zhou X., Wu J., Zhang X., Ding J. (2021). Potential of Long Non-coding RNA KCNQ1OT1 as a Biomarker Reflecting Systemic Inflammation, Multiple Organ Dysfunction, and Mortality Risk in Sepsis Patients. J. Clin. Lab. Anal..

[98] Luo Y., Fu Y., Tan T., Hu J., Li F., Liao Z., Peng J. (2022). Screening of LncRNA-MiRNA-MRNA Coexpression Regulatory Networks Involved in Acute Traumatic Coagulation Dysfunction Based on CTD, GeneCards, and PharmGKB Databases. Oxid. Med. Cell. Longev..

[99] Du J., Chen M., Liu J., Hu P., Guan H., Jiao X. (2019). LncRNA F11-AS1 Suppresses Liver Hepatocellular Carcinoma Progression by Competitively Binding with MiR-3146 to Regulate PTEN Expression. J. Cell. Biochem..

[100] Zhang Z., Yang T., Xiao J. (2018). Circular RNAs: Promising Biomarkers for Human Diseases. EBioMedicine.

[101] Memczak S., Jens M., Elefsinioti A., Torti F., Krueger J., Rybak A., Maier L., Mackowiak S.D., Gregersen L.H., Munschauer M. (2013). Circular RNAs Are a Large Class of Animal RNAs with Regulatory Potency. Nature.

[102] Salzman J. (2016). Circular RNA Expression: Its Potential Regulation and Function. Trends Genet. TIG.

[103] Beltrán-García J., Osca-Verdegal R., Nácher-Sendra E., Cardona-Monzonís A., Sanchis-Gomar F., Carbonell N., Pallardó F.V., Lavie C.J., García-Giménez J.L. (2021). Role of Non-Coding RNAs as Biomarkers of Deleterious Cardiovascular Effects in Sepsis. Prog. Cardiovasc. Dis..

[104] Han B., Chao J., Yao H. (2018). Circular RNA and Its Mechanisms in Disease: From the Bench to the Clinic. Pharmacol. Ther..

[105] Ng W.L., Marinov G.K., Liau E.S., Lam Y.L., Lim Y.-Y., Ea C.-K. (2016). Inducible RasGEF1B Circular RNA Is a Positive Regulator of ICAM-1 in the TLR4/LPS Pathway. RNA Biol..

[106] Shi M., Li Z.-Y., Zhang L.-M., Wu X.-Y., Xiang S.-H., Wang Y.-G., Zhang Y.-Q. (2021). Hsa_circ_0007456 Regulates the Natural Killer Cell-Mediated Cytotoxicity toward Hepatocellular Carcinoma via the MiR-6852-3p/ICAM-1 Axis. Cell Death Dis..

[107] Xiong F., Mao R., Zhang L., Zhao R., Tan K., Liu C., Xu J., Du G., Zhang T. (2021). CircNPHP4 in Monocyte-Derived Small Extracellular Vesicles Controls Heterogeneous Adhesion in Coronary Heart Atherosclerotic Disease. Cell Death Dis..

[108] Liu S., Gao J., Wang S. (2021). HOXA9 Inhibitors Promote Microcirculation of Coronary Arteries in Rats via Downregulating E-Selectin/VCAM-1. Exp. Ther. Med..

[109] Niu F., Liang X., Ni J., Xia Z., Jiang L., Wang H., Liu H., Shen G., Li X. (2022). CircRNA CircFADS2 Is Under-Expressed in Sepsis and Protects Lung Cells from LPS-Induced Apoptosis by Downregulating MiR-133a. J. Inflamm..

[110] Tacke F., Roderburg C., Benz F., Cardenas D.V., Luedde M., Hippe H.-J., Frey N., Vucur M., Gautheron J., Koch A. (2014). Levels of Circulating MiR-133a Are Elevated in Sepsis and Predict Mortality in Critically Ill Patients. Crit. Care Med..

[111] Weyrich A.S., Denis M.M., Schwertz H., Tolley N.D., Foulks J., Spencer E., Kraiss L.W., Albertine K.H., McIntyre T.M., Zimmerman G.A. (2007). MTOR-Dependent Synthesis of Bcl-3 Controls the Retraction of Fibrin Clots by Activated Human Platelets. Blood.

[112] Woulfe D.S. (2010). Akt Signaling in Platelets and Thrombosis. Expert Rev. Hematol..

[113] Yu F., Zhang Y., Wang Z., Gong W., Zhang C. (2021). Hsa_circ_0030042 Regulates Abnormal Autophagy and Protects Atherosclerotic Plaque Stability by Targeting EIF4A3. Theranostics.

[114] Zeng Y., Du W.W., Wu Y., Yang Z., Awan F.M., Li X., Yang W., Zhang C., Yang Q., Yee A. (2017). A Circular RNA Binds To and Activates AKT Phosphorylation and Nuclear Localization Reducing Apoptosis and Enhancing Cardiac Repair. Theranostics.

[115] Cong Y., Li Q., Zhang X., Chen Y., Yu K. (2020). MTOR Promotes Tissue Factor Expression and Activity in EGFR-Mutant Cancer. Front. Oncol..

[116] Hu J., Wang R., Liu Y., Zhou J., Shen K., Dai Y. (2021). Baicalein Represses Cervical Cancer Cell Growth, Cell Cycle Progression and Promotes Apoptosis via Blocking AKT/MTOR Pathway by the Regulation of CircHIAT1/MiR-19a-3p Axis. OncoTargets Ther..

[117] Lu Y.-K., Chu X., Wang S., Sun Y., Zhang J., Dong J., Yan Y.-X. (2021). Identification of Circulating Hsa_circ_0063425 and Hsa_circ_0056891 as Novel Biomarkers for Detection of Type 2 Diabetes. J. Clin. Endocrinol. Metab..

[118] Hu C., Huang L., Gest C., Xi X., Janin A., Soria C., Li H., Lu H. (2012). Opposite Regulation by PI3K/Akt and MAPK/ERK Pathways of Tissue Factor Expression, Cell-Associated Procoagulant Activity and Invasiveness in MDA-MB-231 Cells. J. Hematol. Oncol..

[119] Zhang X., Tang N., Hadden T.J., Rishi A.K. (2011). Akt, FoxO and Regulation of Apoptosis. Biochim. Biophys. Acta BBA-Mol. Cell Res..

[120] Yuan F., Tang Y., Cao M., Ren Y., Li Y., Yang G., Ou Q., Tustumi F., Levi Sandri G.B., Raissi D. (2022). Identification of the Hsa_circ_0039466/MiR-96-5p/FOXO1 Regulatory Network in Hepatocellular Carcinoma by Whole-Transcriptome Analysis. Ann. Transl. Med..

[121] Wang Y., Wang Z., Lu J., Zhang H. (2021). Circular RNA Circ-PTEN Elevates PTEN Inhibiting the Proliferation of Non-Small Cell Lung Cancer Cells. Hum. Cell.

[122] Wang X., Zhang H., Yang H., Bai M., Ning T., Deng T., Liu R., Fan Q., Zhu K., Li J. (2020). Exosome-delivered CircRNA Promotes Glycolysis to Induce Chemoresistance through the MiR-122-PKM2 Axis in Colorectal Cancer. Mol. Oncol..

[123] Zhao X., Tian Z., Liu L. (2021). CircATP2B1 Promotes Aerobic Glycolysis in Gastric Cancer Cells Through Regulation of the MiR-326 Gene Cluster. Front. Oncol..

[124] Chen C., Deng L., Nie D.-K., Jia F., Fu L.-S., Wan Z.-Q., Lan Q. (2020). Circular RNA Pleiotrophin Promotes Carcinogenesis in Glioma via Regulation of MicroRNA-122/SRY-Box Transcription Factor 6 Axis. Eur. J. Cancer Prev..

[125] Jin X., Gao J., Zheng R., Yu M., Ren Y., Yan T., Huang Y., Li Y. (2020). Antagonizing CircRNA_002581–MiR-122–CPEB1 Axis Alleviates NASH through Restoring PTEN–AMPK–MTOR Pathway Regulated Autophagy. Cell Death Dis..

[126] Jin X., Feng C., Xiang Z., Chen Y., Li Y. (2016). CircRNA Expression Pattern and CircRNA-MiRNA-MRNA Network in the Pathogenesis of Nonalcoholic Steatohepatitis. Oncotarget.

[127] Calderone V., Gallego J., Fernandez-Miranda G., Garcia-Pras E., Maillo C., Berzigotti A., Mejias M., Bava F.-A., Angulo-Urarte A., Graupera M. (2016). Sequential Functions of CPEB1 and CPEB4 Regulate Pathologic Expression of Vascular Endothelial Growth Factor and Angiogenesis in Chronic Liver Disease. Gastroenterology.

[128] Garikipati V.N.S., Verma S.K., Cheng Z., Liang D., Truongcao M.M., Cimini M., Yue Y., Huang G., Wang C., Benedict C. (2019). Circular RNA CircFndc3b Modulates Cardiac Repair after Myocardial Infarction via FUS/VEGF-A Axis. Nat. Commun..

[129] Dvorak H.F., Brown L.F., Detmar M., Dvorak A.M. (1995). Vascular Permeability Factor/Vascular Endothelial Growth Factor, Microvascular Hyperpermeability, and Angiogenesis. Am. J. Pathol..

[130] Nakasaki T., Wada H., Shigemori C., Miki C., Gabazza E.C., Nobori T., Nakamura S., Shiku H. (2002). Expression of Tissue Factor and Vascular Endothelial Growth Factor Is Associated with Angiogenesis in Colorectal Cancer. Am. J. Hematol..

[131] Gadomska G., Ziołkowska K., Boinska J., Filipiak J., Rość D. (2019). Activation of TF-Dependent Blood Coagulation Pathway and VEGF-A in Patients with Essential Thrombocythemia. Medicina.

[132] Clauss M., Gerlach M., Gerlach H., Brett J., Wang F., Familletti P.C., Pan Y.C., Olander J.V., Connolly D.T., Stern D. (1990). Vascular Permeability Factor: A Tumor-Derived Polypeptide That Induces Endothelial Cell and Monocyte Procoagulant Activity, and Promotes Monocyte Migration. J. Exp. Med..

[133] Mechtcheriakova D., Wlachos A., Holzmüller H., Binder B.R., Hofer E. (1999). Vascular Endothelial Cell Growth Factor–Induced Tissue Factor Expression in Endothelial Cells Is Mediated by EGR-1. Blood.

[134] Camera M., Giesen P.L.A., Fallon J., Aufiero B.M., Taubman M., Tremoli E., Nemerson Y. (1999). Cooperation Between VEGF and TNF-α Is Necessary for Exposure of Active Tissue Factor on the Surface of Human Endothelial Cells. Arterioscler. Thromb. Vasc. Biol..

[135] Bajzar L., Nesheim M., Morser J., Tracy P.B. (1998). Both Cellular and Soluble Forms of Thrombomodulin Inhibit Fibrinolysis by Potentiating the Activation of Thrombin-Activable Fibrinolysis Inhibitor. J. Biol. Chem..

[136] Wu W., Zou J. (2021). Studies on the Role of CircRNAs in Osteoarthritis. BioMed Res. Int..

[137] Xiao Q., Yin R., Wang Y., Yang S., Ma A., Pan X., Zhu X. (2021). Comprehensive Analysis of Peripheral Exosomal CircRNAs in Large Artery Atherosclerotic Stroke. Front. Cell Dev. Biol..

[138] Larsen J.B., Aggerbeck M.A., Granfeldt A., Schmidt M., Hvas A., Adelborg K. (2021). Disseminated Intravascular Coagulation Diagnosis: Positive Predictive Value of the ISTH Score in a Danish Population. Res. Pract. Thromb. Haemost..

[139] Edwards E., Geng L., Tan J., Onishko H., Donnelly E., Hallahan D.E. (2002). Phosphatidylinositol 3-Kinase/Akt Signaling in the Response of Vascular Endothelium to Ionizing Radiation. Cancer Res..

